# Relationships between gene expression variability, expression levels, and Protein–Protein interactions in mouse and yeast

**DOI:** 10.1371/journal.pone.0352202

**Published:** 2026-06-25

**Authors:** Peter M. Palenchar

**Affiliations:** Villanova University, Department of Chemistry, Radnor, Pennsylvania, United States of America; UC Los Angeles: University of California Los Angeles, UNITED STATES OF AMERICA

## Abstract

Understanding how evolution shapes variability in gene expression is complicated by the interdependence of expression levels, expression variability, and evolutionary rates. Metrics that quantify variability independently of expression would help disentangle these relationships. Previously, a metric termed F* was developed using single-cell RNA-seq data from *Mus musculus*. Here, analyses of single-cell RNA-seq data from *M. musculus* and *Saccharomyces cerevisiae* reveal that the relationship between expression levels and variability is more complex than expected, and F* cannot be fully separated from expression level. Comparisons between single-cell and non-single-cell or simulated bulk experiments show that single-cell data exhibit higher apparent variability for most genes, consistent with contributions from both intrinsic and extrinsic sources. Despite this, the negative relationship between protein-protein interaction connectivity and F* is conserved in both organisms and is also detectable in some non-single-cell datasets, indicating it is not unique to single-cell data or variability. Gene ontology analyses show that, in *M. musculus* across single-cell and non-single-cell datasets, low-F* genes are enriched for translation- and ribosome-associated functions. When single-cell-specific variability is isolated by controlling for non-single-cell contributions, additional enrichment emerges for splicing and spliceosome-associated genes in *M. musculus*, suggesting that genes encoding spliceosome components in *M. musculus* may be under selective pressure to maintain unusually low variation relative to both their expression levels and the variability observed in bulk systems. In no analyses are there over-represented GO terms among the *S. cerevisiae* genes with low F* values.

## Introduction

Variability is inherent in biological systems. Rather than simply minimizing variability, modern research increasingly investigates how organisms manage—and even exploit—gene expression variability. For example, variability can increase phenotypic flexibility and diversity, potentially offering adaptive advantages. As a result, variation in gene expression has been proposed to influence gene evolution [1–5]. However, the relationship between gene evolution and variability in gene expression is complex, due to intertwined correlations among expression levels and variation, evolutionary rates, and other factors [6–9]. A more nuanced understanding of these relationships is essential for clarifying how variability in gene expression levels and evolution interact.

Single-cell (SC) RNA-seq has been instrumental in quantifying gene expression variability. Numerous studies report that genes with high expression levels tend to show lower variation [6,7,10–12]. This negative correlation also holds in bulk (non-single-cell, NSC) datasets [7,13]. To better isolate variability independent of expression levels, recent work has introduced corrected metrics. For instance, local fitting of the mean and standard deviation from RNA-seq data in *S. cerevisiae* and *C. albicans* generated a metric—expression-level adjusted noise (ELAN)—that reduces but does not eliminate the negative correlation between expression level and variability. Interestingly, genes with high ELAN scores often also have high expression levels [7].

Similar analyses of mouse SC RNA-seq data used a third-order polynomial to fit the natural log of variance versus expression level, producing a metric called F*. Unlike CV (coefficient of variance), F* shows no significant correlation with expression level. Despite this, genes with low F* are enriched for GO (gene ontology) terms typically associated with high expression and low variation in expression (e.g., translation and ribosome-related terms) [6,14]. Furthermore, F* correlates positively with Ka/Ks values, suggesting that genes with higher variation in their expression evolve faster—consistent with the broader observation that highly expressed genes tend to evolve more slowly [6,8,9].

Interpreting variability in SC RNA-seq data is further complicated by the contributions of intrinsic and extrinsic variation. Intrinsic variability arises from stochastic fluctuations in molecular processes within cells, while extrinsic variability stems from environmental or technical differences that affect cells unevenly [2,4,15]. SC RNA-seq is often assumed to better capture intrinsic variation, but studies indicate that extrinsic variation remains a confounding factor and is not eliminated in single-cell experiments [16,17]. Moreover, intrinsic and extrinsic variability can share mechanistic explanations both can be enhanced by fluctuations in transcriptional burst size [18], making it difficult to disentangle the two.

Here, to further explore how expression levels and variability interact across systems, we calculated F* for mouse and yeast SC and NSC datasets. We also examined SC-specific variability in an attempt to enrich for intrinsic components of expression variability.

## Methods

### 2.1 RNA-seq data

All the RNA-seq data sets are already published, have been described previously, and include Gene Expression Omnibus repository accession numbers GSE42268, GSM4297055, GSE116246, GSE190764, and GSE190764 [6,7,11,19–21]. The SC mouse data used because they were used previously to determine F* [6]. There are limited yeast single-cell data sets, and for our analysis we require a corresponding NSC data set which further limits the potential data sets. The data used was also used previously to investigate the relationship between gene expression levels and variation [7]. The SC mouse data used is based on 20 cells, while the SC yeast data is based on data from 1,097 cells [11,20]. For previous publications, each data set was processed and filtered to remove lowly expressed genes [6,7,11,19,20,22]. The *S. cerevisiae* NSC RNA-seq data was available as FPM (Fragments Per Million), which does not consider gene lengths. To make it more comparable to the other data sets that are available as FPKM (Fragments Per Kilobase of transcript per Million mapped reads), the FPM values were divided by gene lengths. All analyses involving gene expression levels are based on the mean gene expression level for each gene. Analyses of SC RNA-seq data sets are based on the mean gene expression level for a gene across all the cells.

### 2.2 Determining F*

F* was determined as described previously [6], with modifications to use the CV or the difference in CV values in some cases. Briefly, different order polynomial equations were fit to the natural log of the mean expression levels and variance, CV values, or differences in CV values. For each gene, the polynomial equation was used to calculate an expected variance, CV, or difference in the CV values based on the gene’s mean expression level. The F* value for the gene is the actual variance, CV, or difference in the CVs/the expected variance, CV, or difference in the CVs. The correlation between the F* and mean gene expression levels were determined. The F* from the lowest order polynomial that resulted in a p-value > 0.05 for the correlation were used. For the SC mouse RNA-seq data initially, all the genes previously used were used to determine F* [6], however all the genes were not assigned PPI hub scores. To simplify the analyses when analyzing non-single-cell mouse data and determining single-cell specific variability, only genes that were assigned PPI were used.

### 2.3 PPI hub scores

PPI hub scores were calculated using the hub_score() function in the igraph R package, which estimates node centrality from the network adjacency matrix rather than simply counting interaction partners. Mouse protein-protein interaction (PPI) and pathway hub scores were previously determined using the *igraph* package in R. S. *cerevisiae* PPI hub scores were calculated using the same method from protein-protein interaction data extracted from BioGRID [6,23–27]. To maintain comparability with the previous mouse study, PPI hub scores from Barroso *et al.* were used in all analyses [6].

For *S. cerevisiae*, hub scores were computed both from all reported physical interactions in BioGRID and from a filtered set including only interactions confirmed by more than one publication. Among genes with single-cell RNA-seq data, requiring interactions to occur more than once reduced the dataset by 349 genes. The two sets of yeast PPI hub scores were strongly correlated (0.500; p-value < 0.0002). Both versions of the hub scores were positively correlated with gene expression levels (0.285 for all interactions vs. 0.295 for confirmed interactions; p-values < 0.0002) and negatively correlated with F* values derived from the SC data (−0.209 and −0.094; p-values < 0.0002).

Genes excluded by requiring multiple supporting publications had significantly lower median expression levels than those retained (11.55 ± 12.69 vs. 7.52 ± 7.69; p-values < 0.0002). Given the overall similarity between the two hub score sets but the expression bias introduced by filtering, all subsequent analyses used the full set of BioGRID interactions.

However, the mouse genes with PPI hub scores are not representative of the general population of mouse genes (see Results for more details), so after the initial analyses, analysis focused on the mouse genes with PPI hub scores and only those genes were used to determine F*. For *S. cerevisiae*, PPI hub scores were not determined for only 9 of the genes in the RNA-seq data, the presence of the 9 genes did not affect the overall results (data not presented), and all the genes in the RNA-seq data were used to determine F*.

For the mouse data, only 38 genes have higher CV values in the NSC data than the SC data. In the yeast data, only 2 genes have a CV that is lower in the SC RNA-seq data than the NSC RNA-seq data, so the differences in the CVs were used to determine the F* of the SC-specific variability for the yeast data instead of the variance. Genes with lower CVs in the SC were not included.

### 2.4 Identification of Gene Subsets

To make the results herein comparable to the previous study, genes with high or low values were based on the top or bottom 10% [6]. A randomization method was used to determine if there was a larger overlap between groups (e.g., genes with high expression levels and F* levels) where the number of genes that overlap in the actual case were compared to the overlap of randomly selected sets of the same number of genes as described previously [7].

### 2.5 Identifying single-cell specific variability

In an attempt to minimize the contribution of intrinsic transcriptional variability, we employed a pseudo-bulk sampling strategy. Cells were randomly divided into two groups of equal size, and the coefficient of variation (CV) of gene expression was calculated between the two group means. This procedure was repeated 5,000 times, and the median CV across replicates was used as the final estimate. Pooling gene expression measurements across cells reduces stochastic cell-specific fluctuations (intrinsic variation), which are uncorrelated among cells, while maintaining shared extrinsic influences [2,3]. Because the intrinsic component of variance decreases as the number of cells per group increases, pseudo-bulk averaging yields an estimate that likely heavily reflects extrinsic and systematic variation [28,29].

### 2.6 General statistics and other analyses

All statistics and analyses were conducted with R version 4.0.2 [30]. To be consistent with the methods used by Barroso et al., correlations were computed using the Kendall method [6]. P-values were determined via a randomization procedure [31].

We assessed the relationship between gene expression and F* using three complementary approaches: global rank concordance (Kendall’s correlations) [32], change-point analysis to identify structural shifts, and visualization of local trends using segment-wise linear fits. To remain consistent with the correlation analyses and to mitigate the influence of outliers, change-point analyses were also performed on ranked values. Change-point analyses were conducted using the bcp package [33] and further analyses focused on the region with the highest posterior probability of a change point; other potential shifts were not examined. Kendall’s correlation measures pairwise rank concordance and does not directly reflect the magnitude or direction of average trends, but the direction of the segment-wise slopes were consistent with the corresponding rank correlations.

Correlations were compared using the cocor package [34]. Gene Ontology (GO) analyses were conducted using the goseq, org.Mm.e.g.,db, and org.Sc.sgd.db packages and Bioconductor [35,36]. GO annotations were analyzed for the three Gene Ontology domains (Biological Process, Molecular Function, and Cellular Component). The background genes for GO analysis were limited to the genes in the analysis (e.g., genes in the RNA-seq data with PPI hub scores) and conducted using the Benjamini–Hochberg false discovery rate correction as described previously [7].

## Results

### 3.1 Determining F* and its independence from gene expression levels

Previously, SC RNA-seq data from mouse ESG1 cells were used to develop F*, a metric of gene expression variability designed to be independent of expression level. Genes with low F* were enriched for characteristics typically associated with high expression—for example, GO terms related to translation and ribosome function [6]. To confirm this, GO analyses of the genes that are highly expressed in the SC ESG1 RNA-seq data set were conducted and showed that highly expressed genes were enriched for translation- and ribosome-associated GO terms (Supplemental Table 1 in S1 File), overlapping with those enriched among genes with low F* (Supplemental Tables 1 and 2 in S1 File).

F* has also been found to correlate with PPI and mean pathway hub scores [6], suggesting that genes with more biological interactions at the protein level exhibit lower expression variability. Given that genes with low F* and high expression share GO terms, we examined the relationship between gene expression and PPI hub scores. Of 13,660 genes analyzed for expression, PPI hub score data were available for 5,553 genes. These genes tend to be more highly expressed than those lacking hub scores (Table 1).

**Table 1:** Comparisons of the median gene expression levels and the median absolute deviation based on the mouse SC RNA-seq data of different sets of genes. Tests were done to determine if the genes with PPI and mean pathway hub scores have different median expression levels than all the genes.

Genes used in analysis	Number of genes	Median gene expression values	p-value
All	13,660	9.39 ± 12.0	–
Genes whose product have a PPI hub score	5,553	14.37 ± 18.0	<0.0002
Genes whose product have a mean pathways hub score	4,454	13.61 ± 17.1	<0.0002

Gene expression levels were positively correlated with PPI hub scores, whereas CV values were negatively correlated (Supplemental Table 3 in S1 File). Examining F* among genes with PPI hub scores revealed a weak but significant positive correlation with expression (0.026; p-value = 0.006), while a similar analysis using pathway hub scores showed a correlation near zero and not statistically significant (Supplemental Table 4 in S1 File).

To further understand differences between PPI hub genes and the full gene set, GO enrichment analysis was performed. Genes with PPI hub scores were overrepresented in binding, protein complex, and regulatory categories (Supplemental Table 5 in S1 File) and underrepresented in membranes, protein glycosylation, and tRNA processing (Supplemental Table 6 in S1 File). Enrichment of binding- and protein complex-associated functions is not surprising, as proteins with many interaction partners are expected to participate in intermolecular interactions. An underrepresentation of membrane-associated proteins may partially reflect known biases in some PPI detection methods, which can miss interactions involving membrane proteins [37,38].

Next, NSC RNA-seq data from G1 ES mouse cells were examined. For simplicity, the analysis was limited to genes with available PPI hub scores. Gene expression levels and CVs were strongly correlated between the SC and NSC datasets (0.610 and 0.174, respectively; both p-value < 0.0002). F* was calculated from the NSC data (Supplemental Table 8 in S1 File), using a first-order polynomial fit. The resulting F* values showed a weak positive correlation with gene expression levels (0.017; p-value = 0.1644), which was not statistically significant. Notably, F* from the NSC data was correlated with F* from the SC data (0.05; p-value < 0.0002), indicating that single-cell and non-single-cell variability are related even after a correction for gene expression levels.

To further explore the relationship between gene expression level and variability in yeast, we applied the same analyses to SC RNA-seq data from *S. cerevisiae*. A second-degree polynomial fit effectively removed most of the correlation between F* and mean gene expression (0.0009; p-value = 0.476; Supplemental Table 9 in S1 File). As reported previously (Palenchar and DeStefanis 2022), mean gene expression and CVs are strongly negatively correlated (−0.706; p-value < 0.0002), and F* is positively correlated with CV (0.293; p-value < 0.0002). For comparison, in the mouse SC data, F* and CV are also positively correlated (0.328; p-value < 0.0002), and CV is strongly negatively correlated with gene expression (−0.675; p-value < 0.0002). For both yeast and mouse SC datasets, GO terms enriched among highly expressed genes include categories associated with the ribosome, translation, and peptide bond formation (Supplemental Tables 1 and 10 in S1 File). However, in yeast, genes with low F* are not enriched for any GO terms, in contrast to the mouse results.

Next, NSC yeast data were analyzed alongside the SC dataset. Gene expression levels and CVs remained well correlated between SC and NSC data (0.665 and 0.308, respectively; both p-values < 0.0002). F* was calculated using a first-order polynomial fit, resulting in a correlation with gene expression near zero and not statistically significant (0.002; p-value = 0.452; Supplemental Table 11 in S1 File). Despite differences in the fitting procedure, F* from the SC and NSC yeast datasets are correlated (0.273; p-value < 0.0002), again indicating that variability measured in single-cell and bulk data are related. Like the SC data, GO analysis identified no over-represented GO terms among the genes with low F* values.

### 3.2 SC-specific variability

If NSC RNA-seq studies average expression across many cells, information about cell-to-cell variability is reduced, and differences between samples may primarily reflect extrinsic sources of variation. In contrast, variability observed in SC RNA-seq data reflects contributions from both intrinsic and extrinsic sources [16,17]. Subtracting variability from NSC data from that observed in SC data therefore could provide a measure of SC-specific variability, which may be enriched for cell-to-cell variation relative to SC or NSC measurements alone.

Comparing the CV between SC and NSC G1- ES cell mouse datasets show that, for most genes, CV values are higher in the SC data. Only 38 genes exhibit a negative difference, and the mean SC-NSC CV difference is 1.28 ± 0.95. The higher CVs observed for most genes in SC data are consistent with SC measurements capturing additional sources of variability that are reduced in NSC experiments.

These SC-NSC CV differences were used to calculate F* (Supplemental Table 12 in S1 File). Even when fitting up to a tenth-order polynomial, the resulting F* values retained a weak but statistically significant relationship with gene expression (−0.017; p-value = 0.0186). To assess how these values relate to F* derived from SC or NSC data alone, pairwise correlations were calculated. The SC-NSC-derived F* values were strongly correlated with SC-based F* (0.796; p-value < 0.0002) but showed no meaningful correlation with NSC-derived F* (0.014; p-value = 0.0676).

To further approximate SC-specific variability, cells from the SC mouse dataset were repeatedly partitioned into two groups, and CVs were calculated for each group. For every gene, the mean CV derived from grouped cells was lower than the CV calculated from true single-cell measurements. The mean ratio of single-cell CV to median CV from the grouped data was 3.80 ± 0.23, consistent with grouping cells reducing cell-to-cell variability relative to single-cell measurements. F* was calculated from the difference between the CV of the SC data and the median CV of the grouped data (Supplemental Table 13 in S1 File). Using a third-order polynomial fit, this version of F* showed little overall correlation with mean gene expression levels (−0.0038; p-value = 0.329) and was positively correlated with F* from the SC data (0.967; p-value < 0.0002) and from the NSC data (0.056; p-value < 0.0002).

Analysis of genes with low F* from both methods of estimating SC-specific variability indicated enrichment for genes involved in translation and the ribosome, similar to the SC-derived F*. In both cases, genes involved in splicing and the spliceosome were also enriched, suggesting that expression of splicing factors may be especially constrained to low SC-specific variability (Supplemental Tables 14 and 15 in S1 File).

Only two analyzed yeast genes have a CV that is higher in the NSC data than in the SC data, which is again consistent with the idea that SC data contain both intrinsic and extrinsic variability. The mean SC–NSC CV difference is 5.23 ± 3.20. F* based on the differences in the CV of the SC and NSC data was generated using a second-order polynomial fit (correlation with mean gene expression = 0.0006; p-value = 0.475) (Supplemental Table 16 in S1 File). The resulting F* values are positively correlated with the SC-derived F* (0.909; p-value < 0.0002) and with the NSC-derived yeast F* (0.189; p-value < 0.0002).

As with the mouse data, the SC yeast data were randomly partitioned, and CVs were calculated for each grouped dataset. For every gene, the mean grouped CV was lower than the corresponding SC CV, and the mean difference between SC CVs and grouped CVs was 5.21 ± 3.17. F* was then calculated from the difference between the SC CV and the grouped CV (Supplemental Table 17 in S1 File). Using a second-order polynomial fit, this version of F* showed little overall correlation with mean gene expression levels (0.0005; p-value = 0.4892) and was positively correlated with the SC-derived (0.998; p-value < 0.0002) and NSC-derived (0.273; p-value < 0.0002) F* values. In contrast to the mouse analysis, however, yeast genes with low F* were not enriched for any GO terms.

### 3.3 Testing the Independence of Gene Expression and F*

For F* derived from differences in CVs between the SC and NSC mouse data, the correlation between gene expression levels and F* did not approach zero and remained statistically significant. In contrast, for the other analyses, correlations between F* and gene expression were close to zero and not statistically significant. Previously, F* was considered to be a measure of variability independent of gene expression level [6]. However, the similarity in enriched GO terms between genes with low F* and those with high expression in the SC mouse data suggests that Kendall’s correlation alone may not fully capture their relationship. To further evaluate this relationship in cases where the overall correlation was near zero, genes with high and low F* and with high and low expression levels were identified for each analysis, and overlaps among these categories were assessed (Supplemental Tables 18-26 in S1 File).

In every case, at least one combination of high/low F* and high/low expression showed a non-random overlap. These patterns supported a more complex relationship between F* and gene expression than indicated by simple rank correlations. For example, in the mouse SC dataset, genes with high F* were significantly under-represented among lowly expressed genes (Supplemental Table 19). Interestingly, the reverse did not hold: genes with low F* were not overrepresented among highly expressed genes. The absence of a reciprocal enrichment among low-F* and high-expression genes is therefore consistent with the lack of a simple negative correlation between F* and gene expression.

To further investigate the relationship between F* and gene expression levels in the cases where the correlation was close to zero, a change-point analysis was performed to identify whether the association between these variables shifts across the range of gene expression levels. If F* were truly independent of gene expression, no systematic pattern would be expected, and the analysis would be unlikely to detect any point with a high probability of a change in relationship. For the single-cell mouse data, the analysis revealed a greater than 81% probability of a change occurring near the gene ENSMUSG00000041064, which is ranked 1,404th by expression level. This suggests a potential shift in the relationship between F* and gene expression at this point in the ranked data. To further evaluate this shift, correlation coefficients between F* and mean gene expression were calculated separately for the 1,399 genes with the lowest expression and for the genes ranked 1,409 and higher (i.e., those with higher expression levels) (Table 2).

**Table 2:** Correlation and p-values between gene expression levels and F* based on the SC mouse ESG1 RNA-seq data.

Genes	Correlation between ranks of gene expression and F*
All genes	−0.003 (p-value = 0.2572)
Genes with gene expression values ranked from 1−1,399	0.139 (p-value < 0.0002)
Genes with gene expression values ranked from 1,409−13,660	−0.029 (p-value < 0.0002)

**Fig 1:**
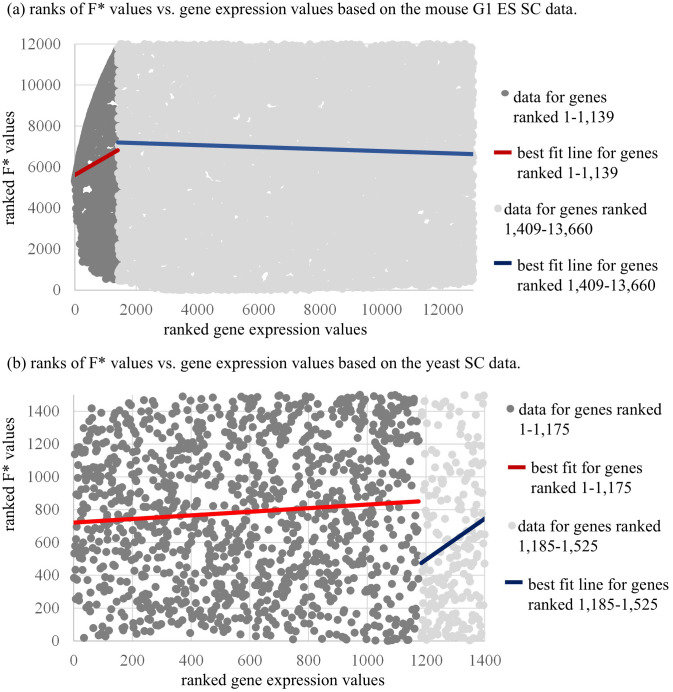
Relationship between the ranks of F* and the ranks of mean gene expression values from mouse ESG1 SC RNA-seq data (a) and yeast SC RNA-seq data (b). Data are segmented based on change-point analysis, with best-fit lines are shown on either side of the detected change point for visualization.

Kendall’s correlations between F* and mean expression were calculated separately for the 1,399 lowest-expressed genes and the remaining genes (ranked 1,409–13,660). Among low-expression genes, F* showed a weak but significant positive correlation with expression (0.139, p-value < 0.0002), whereas the higher-expression group showed a weak negative correlation (−0.029, p-value < 0.002) (Table 2). These correlations differed significantly (p-value < 0.01), supporting distinct patterns across expression ranges. Best-fit lines were used to visualize the relationship between F* and expression in the two subsets of genes (Fig 1).

F* for the SC mouse ESG1 data was originally derived using a third-degree polynomial fit to model the relationship between variance and gene expression levels. To further reduce the correlation between variability and expression across all genes—including those with low expression—higher-degree polynomial fits were tested. However, even with fits up to the 13th degree, the resulting values remained positively correlated with expression among lowly expressed genes and negatively correlated among highly expressed genes (Supplemental Table 27 in S1 File). These extended fits failed to eliminate the dependency of the variability metric on gene expression level for most genes.

For the single-cell yeast data, a change-point analysis identified a shift in the relationship between F* and gene expression values near the 1,180th ranked gene (YOR237W), with >86% confidence (Table 3). For genes ranked below 1,175 in mean expression, the correlation between F* and expression is 0.062 (p-value = 0.0014), while for those ranked above 1,185, the correlation is 0.22 (p-value < 0.0002). The difference in these correlations is significant (p-value = 0.0108). Best fit lines based on the ranked F* and gene expression values support this change in relationship (Fig 1).

**Table 3:** Results of the change-point analysis for the relationship between ranked gene expression levels and F* values. The rank corresponding to the greatest change point probability and its associated probability are reported. To avoid local effects at the change point dominating both correlations, genes with ranks close to the change point were excluded. Correlations between gene expression and F* were then calculated separately for genes ranked above and below this buffered region. Correlation coefficients are shown with p-values in parentheses.

Data analyzed	Area of greatest change point	Probability	Correlation from lowly ranked genes	Correlation from highly ranked genes
SC mouse with PPI hub-scores	658	>95%	0.0074 (0.0022)	−0.0006 (0.471)
NSC G1 mouse	5,375	>98%	−0.26 (0.0028)	0.11 (0.062)
Difference between SC mouse data and grouped SC mouse data	3,467	>90%	0.015 (0.081)	0.034 (0.0262)
Difference between SC and bulked SC mouse data	1,487	>87%	0.034 (0.0288)	0.015 (0.0756)
SC yeast	1,180	>86%	0.062 (0.0014)	0.22 (< 0.0002)
NSC yeast	1,385	>87%	0.024 (0.0846)	0.170 (0.0012)
Difference between SC and NSC yeast data	1,125	>95%	0.049 (0.0086)	0.110 (0.0004)
Difference between SC and bulked SC yeast data	460	>95%	0.008 (0.3986)	0.010 (0.3092)

Change-point analysis between the other F* and gene expression levels all indicate areas with high probabilities of changes in the relationship between F* and gene expression levels (Table 3). As with the SC mouse and yeast data, although the overall correlation between F* and gene expression levels is near zero and not statistically significant, this does not imply that no relationship exists between these variables. Instead, different subsets of genes display distinct patterns in the relationship between gene expression and F*, indicating that population-wide analyses can obscure important trends within subgroups.

To visualize these patterns, best-fit lines were generated for the relationship between F* rank and gene expression rank for the two expression-level subsets (Fig 1 and Figures S1 and S2 in File S1 Fig and S2 Fig).

### 3.4 Relationship Between F* and PPI hub scores

Although the analysis indicates that calculating F* does not completely eliminate the relationship between gene expression level and variation, the association is substantially reduced at the global level. Given this reduction—and because previously F* was calculated using genes without PPI hub scores^6^—it remains important to determine whether a global relationship persists between expression variability (F*) and PPI hub scores. In other words, even after reducing the dependence of F* on expression level and restricting the analysis to genes with PPI hub scores, it remains unclear whether protein-protein interaction connectivity is linked to variability in gene expression.

For every F* measure examined, except for the mouse NSC ESG1 dataset, the correlation between F* and PPI hub scores is negative (Table 4), indicating that genes with more protein–protein interactions tend to exhibit lower variability. The absence of a significant correlation in the mouse NSC ESG1 data raised the question of whether the relationship in mouse is specific to intrinsic variability. To test this directly, an independent mouse NSC dataset (adult neural stem cells) was analyzed and F* was recalculated (Supplemental Table 28 in S1 File) [21]. The resulting F* values were negatively correlated with PPI hub scores (−0.035, p-value = 0.0002), indicating that the negative association between F* and PPI hub scores is not restricted to SC RNA-seq data in mouse.

**Table 4:** Correlations and p-values for the correlation of PPI hub scores to F* determined different ways.

Data/genes analyzed	Correlation	p-value
All genes with data in SC mouse data	−0.081	<0.0002
SC mouse data for genes with PPI hub scores	−0.105	< 0.0002
ESG1 NSC mouse data for genes with PPI hub scores	0.004	0.3082
difference between gene expression levels in SC and grouped single ESG1 mouse data	−0.106	< 0.0002
SC yeast data	−0.209	< 0.0002
NSC yeast data	−0.169	< 0.0002
difference between gene expression levels in SC and non-single yeast data	−0.208	< 0.0002
difference between gene expression levels in SC and grouped single yeast data	−0.209	< 0.0002

## Discussion

This study highlights the challenges of generating metrics for variability in gene expression that are independent of gene expression levels. Even when higher-order polynomial fits are used, relationships between F* and expression remain difficult to eliminate entirely. While these correlations can be close to zero, the persistence of detectable relationships suggests that intrinsic properties of gene expression inevitably intertwine with mean expression levels, limiting the extent to which purely expression-independent measures of variability can be achieved using polynomial fits.

Previously, a negative correlation between F* and PPI hub scores was reported but did not account for the fact that, among genes with PPI hub scores, F* and gene expression levels are themselves correlated [6]. Recalculating F* using only genes with PPI hub scores produced an F* independent of gene expression levels (based on Kendall’s correlation) (Section 3.1). The results remain consistent with a negative relationship between gene expression variability and protein-protein interaction connectivity even after accounting for gene expression levels at the proteome-wide level and attempting to isolate SC-specific variability.

Further, the results suggest that for most genes in mouse and yeast SC gene expression data is more complex and higher than NSC data as the CV for most genes is higher in SC data than NSC data. To identify SC-specific expression variability, two complementary approaches were used: (1) subtracting the CV from NSC data from the CV measured in SC experiments, and (2) grouping SC data to mimic NSC experiments and then calculating a CV difference. The mouse F* calculated by directly subtracting the NSC CV from the SC CV was an exception, but in all other cases, estimates of SC-specific variability were well correlated with F* values derived from NSC data. This consistent correlation suggests that intrinsic and extrinsic variability are not independent phenomena but rather are mechanistically linked.

Despite these overall similarities, yeast and mouse differed in their biological enrichments of genes with low F*. Yeast genes with low F* consistently showed no GO term enrichments, whereas mouse genes with low F* were strongly enriched for terms associated with translation and ribosomal function. Strikingly, both approaches to isolating SC-specific variability in the mouse data also identified enrichment for genes involved in splicing and the spliceosome. This pattern suggests that in multicellular contexts, regulation of translational and RNA-processing machinery may require especially low variability when accounting for expression levels, potentially to safeguard the fidelity of protein production and mRNA maturation.

Notably, in both organisms and across SC and NSC datasets, highly expressed genes showed enrichment for broadly similar GO terms. Thus, while the biological processes associated with high expression appear conserved, the processes associated with low F* diverge. However, these differences should be interpreted cautiously, as they may reflect differences in experimental conditions or dataset composition (e.g., variation in cell-cycle representation) rather than intrinsic organismal differences.

## Supporting information

S1 File Supplemental Tables 1–28. (XLSX)

S1 Fig Ranks of F* versus gene expression values for (a) SC G1 ES mouse cell data for genes with PPI hub scores, (b) NSC ES mouse data, and (c) F* derived from differences between SC and grouped SC CVs. Change points and corresponding Kendall correlations for each segment are reported in Table 2. In each panel, darker gray circles represent genes with expression values ≤5 ranks below the detected change point, with the dark red line indicating the corresponding best-fit line. Light gray circles represent genes with expression values ≥5 ranks above the change point, with the dark blue line indicating the corresponding best-fit line.
(TIF)

S2 Fig Ranks of F* versus gene expression values for (a) yeast NSC mouse data, (b) F* derived from differences between SC and NSC CVs, and (c) F* derived from differences between SC and grouped SC CVs. Change points and corresponding Kendall correlations for each segment are reported in Table 2. In each panel, darker gray circles represent genes with expression values ≤5 ranks below the detected change point, with the dark red line indicating the corresponding best-fit line. Light gray circles represent genes with expression values ≥5 ranks above the change point, with the dark blue line indicating the corresponding best-fit line.
(TIF)
