# Supplementary figures and images for: Relationships between gene expression variability, expression levels, and Protein–Protein interactions in mouse and yeast
Source: PLoS One. 2026 Jun 25;21(6):e0352202. doi: 10.1371/journal.pone.0352202 (PMC13298762; doi:10.1371/journal.pone.0352202)

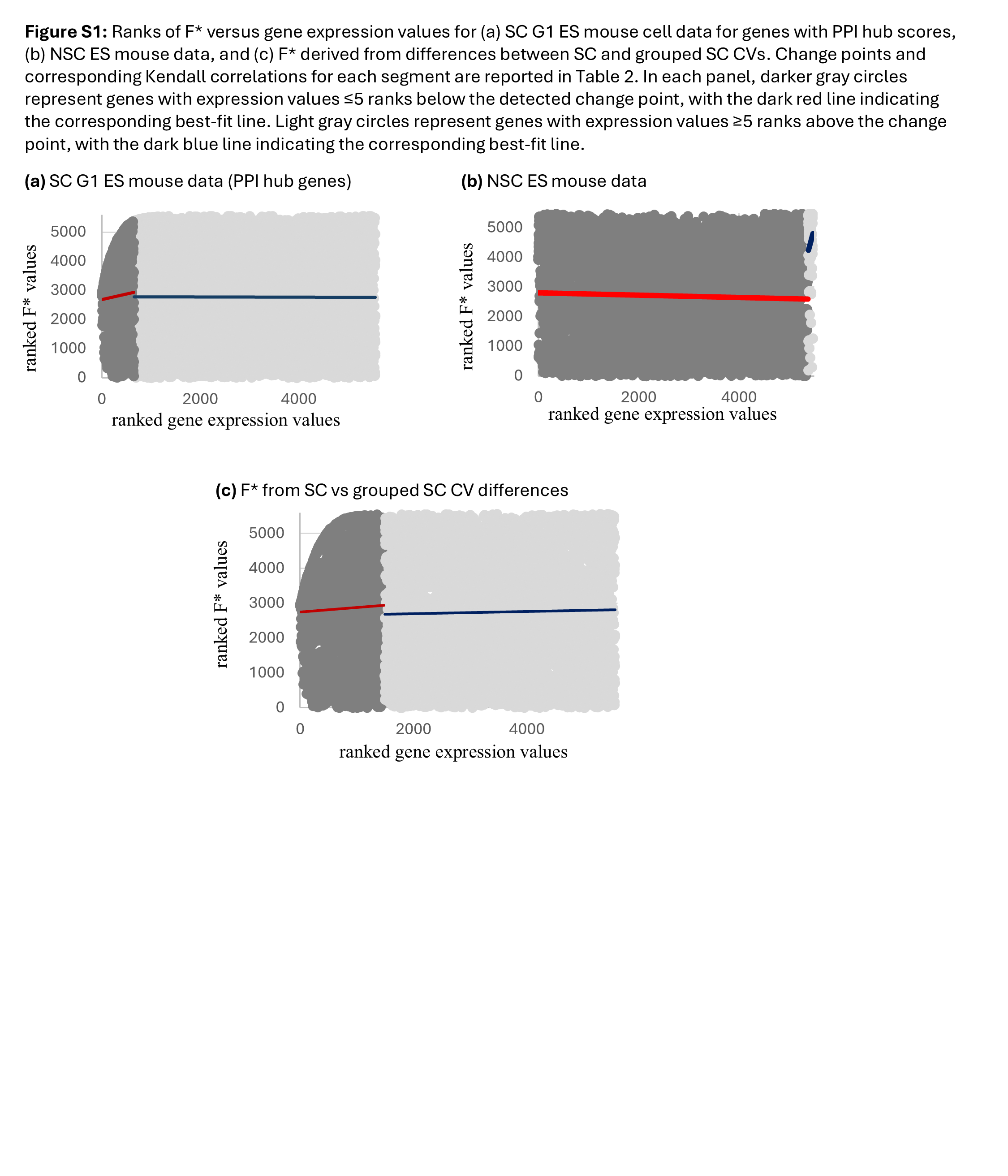

Supplement: S1 Fig — Change points and corresponding Kendall correlations for each segment are reported in Table 2. In each panel, darker gray circles represent genes with expression values ≤5 ranks below the detected change point, with the dark red line indicating the corresponding best-fit line. Light gray circles represent genes with expression values ≥5 ranks above the change point, with the dark blue line indicating the corresponding best-fit line. (TIF) [file pone.0352202.s002.tif]

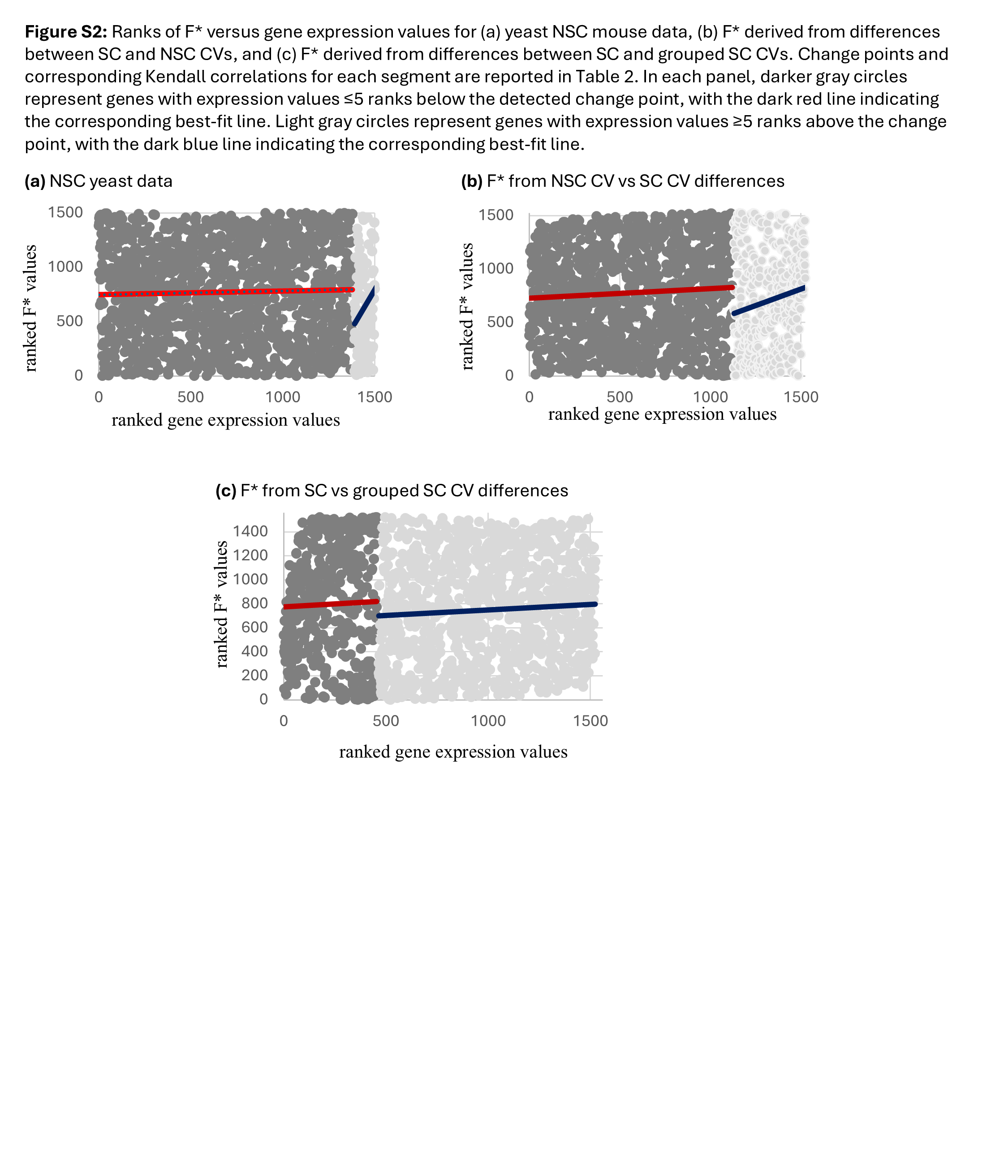

Supplement: S2 Fig — Change points and corresponding Kendall correlations for each segment are reported in Table 2. In each panel, darker gray circles represent genes with expression values ≤5 ranks below the detected change point, with the dark red line indicating the corresponding best-fit line. Light gray circles represent genes with expression values ≥5 ranks above the change point, with the dark blue line indicating the corresponding best-fit line. (TIF) [file pone.0352202.s003.tif]
